# The effects of seasonal human mobility and *Aedes aegypti* habitat suitability on Zika virus epidemic severity in Colombia

**DOI:** 10.1371/journal.pntd.0012571

**Published:** 2024-11-06

**Authors:** Brandon Lieberthal, Brian Allan, Sandra De Urioste-Stone, Andrew Mackay, Aiman Soliman, Shaowen Wang, Allison M. Gardner

**Affiliations:** 1 College of Natural Sciences, Forestry, and Agriculture, University of Maine, Orono, Maine, United States of America; 2 School of Integrative Biology, University of Illinois, Urbana-Champaign, Illinois, United States of America; 3 Illinois Natural History Survey, Champaign, Illinois, United States of America; 4 National Center for Supercomputing Applications, University of Illinois, Urbana-Champaign, Illinois, United States of America; 5 College of Liberal Arts and Sciences, University of Illinois, Urbana-Champaign, Illinois, United States of America; University of California Davis School of Veterinary Medicine, UNITED STATES OF AMERICA

## Abstract

The Zika virus epidemic of 2015–16, which caused over 1 million confirmed or suspected human cases in the Caribbean and Latin America, was driven by a combination of movement of infected humans and availability of suitable habitat for mosquito species that are key disease vectors. Both human mobility and mosquito vector abundances vary seasonally, and the goal of our research was to analyze the interacting effects of disease vector densities and human movement across metapopulations on disease transmission intensity and the probability of super-spreader events. Our research uses the novel approach of combining geographical modeling of mosquito presence with network modeling of human mobility to offer a comprehensive simulation environment for Zika virus epidemics that considers a substantial number of spatial and temporal factors compared to the literature. Specifically, we tested the hypotheses that 1) regions with the highest probability of mosquito presence will have more super-spreader events during dry months, when mosquitoes are predicted to be more abundant, 2) regions reliant on tourism industries will have more super-spreader events during wet months, when they are more likely to contribute to network-level pathogen spread due to increased travel. We used the case study of Colombia, a country with a population of about 50 million people, with an annual calendar that can be partitioned into overlapping cycles of wet and dry seasons and peak tourism and off tourism seasons that drive distinct cyclical patterns of mosquito abundance and human movement. Our results show that whether the first infected human was introduced to the network during the wet versus dry season and during the tourism versus off tourism season profoundly affects the severity and trajectory of the epidemic. For example, Zika virus was first detected in Colombia in October of 2015. Had it originated in January, a dry season month with high rates of tourism, it likely could have infected up to 60% more individuals and up to 40% more super-spreader events may have occurred. In addition, popular tourism destinations such as Barranquilla and Cartagena have the highest risk of super-spreader events during the winter, whereas densely populated areas such as Medellín and Bogotá are at higher risk of sustained transmission during dry months in the summer. Our research demonstrates that public health planning and response to vector-borne disease outbreaks requires a thorough understanding of how vector and host patterns vary due to seasonality in environmental conditions and human mobility dynamics. This research also has strong implications for tourism policy and the potential response strategies in case of an emergent epidemic.

## Author summary

The Zika virus pandemic of 2015–16, which caused over 1 million human cases in the Caribbean and Latin America, was driven by a combination of movement of infected humans and presence of mosquito species, such as *Aedes aegypti*, that carry the disease. Both human mobility and mosquito vector densities vary seasonally, and the goal of our research was to analyze the interacting effects of disease vector presence and human movement across metapopulations on disease transmission intensity and the probability of super-spreader events. Specifically, we tested the hypotheses that 1) regions with the highest probability of mosquito presence will have more super-spreader events during dry months, when mosquitoes are predicted to be more abundant, 2) regions reliant on tourism industries will have more super-spreader events during wet months, as they are more likely to contribute to network-level pathogen spread due to increased travel. We used the case study of Colombia, a nation with a population of about 50 million people, whose annual calendar can be partitioned into overlapping cycles of wet and dry seasons and tourism and off-tourism seasons that drive distinct cyclical patterns of mosquito occurrence and human movement. Our approach combined spatial modeling of mosquito presence with network modeling of human mobility. We used a Maximum Entropy model to estimate the spatially and temporally varying habitat suitability for Aedes aegypti, the primary mosquito vector of Zika virus in Colombia, in terms of environmental factors such as temperature, precipitation, and vegetation coverage and human factors indicating land use and development, socioeconomic status, and prevalence of standing water that provides key habitat for juvenile mosquitoes. We also used airline travel data to develop metapopulation patch network models of the 1,123 municipalities of Colombia for each month of 2015 and 2016. We combined our vector habitat suitability and human mobility network models to simulate the Zika virus epidemic if it had begun in each month of 2015. Our results show that whether the first human case was introduced to the network during the wet vs. dry season and during the tourism vs. off-tourism season profoundly affects the severity and trajectory of the epidemic. For example, Zika virus was first detected in Colombia in October of 2015. Had it originated in January, a dry season month with high rates of tourism, it likely could have infected up to 30% more individuals and up to 40% more super-spreader events may have occurred. In addition, popular vacation spots such as Barranquilla and Cartageña have the highest risk of super-spreader events during the winter, whereas densely populated areas such as Medellín and Bogotá are at higher risk of sustained transmission during dry months in the summer. Our research demonstrates that public health planning and response to vector-borne disease outbreaks requires a thorough understanding of how vector and host patterns vary due to seasonality in environmental conditions and human mobility dynamics.

## Introduction

Zika virus is a member of the family Flaviviridae that garnered considerable attention due to causing a widespread outbreak in the Americas since 2015 [1]. Zika virus was first discovered in Uganda in 1947, but the first recognized epidemic of significant scale occurred in 2007 in Gabon, with 4312 known cases [2]. Range expansion of Zika virus was observed throughout the Pacific region, including an outbreak in French Polynesia in 2013. Subsequently, Zika virus was introduced to Brazil [3–5], where human Zika cases were first confirmed in early 2015 [6]. Zika quickly disseminated to other countries in South and Central America, the Caribbean, and Mexico, followed by locally-acquired cases in the US states of Florida and Texas in 2016 [7]. The 2015–16 outbreak was responsible for an estimated 1.5 million human cases throughout the Western hemisphere.

Movement of infected (often asymptomatic) humans is thought to be primarily responsible for the spread of multiple arthropod-borne viruses (arboviruses), also including chikungunya virus and dengue fever [8,9], and international travel is believed to have played a crucial role in the emergence of Zika virus across the globe [10–12]. While the precise mechanism by which Zika virus was introduced to the Americas is unknown, phylogenetic analysis indicates it was likely due to the introduction of the virus by an infected human traveler, potentially arriving in Brazil as early as May 2013 [13]. Human mobility likely plays a critical role not only in the movement of arboviruses including Zika to new locations [14,15], but also in their establishment and persistence within their introduced ranges. However, difficulty characterizing human movement has impeded inclusion of human mobility as a parameter in risk models for arbovirus transmission. Several data sources are available to capture human movements across different spatial and temporal scales, including internal migration records [16], air travel records [17], phone call records [15], location-based social media data and other sources of microdata [18,19]. Incorporating these mobility data in studying disease transmission enhances the understanding of disease transmission and increases the accuracy of prediction models [20].

The environmental suitability for the introduction of Zika virus to the Americas was seeded years earlier by the globalization of mosquitoes that serve as the primary vectors of the virus, mainly *Aedes aegypti* (yellow fever mosquito) and *Aedes albopictus* (Asian tiger mosquito). These species thrive in urban ecosystems, both through adaptations to complete juvenile development in artificial aquatic container habitats typically found at higher abundances in urban environments, and a preference for blood feeding from humans and other mammals [21]. Populations of both species can exhibit recurrent fluctuations in abundance related to seasonal changes in rainfall and temperature and their effects on human behaviors (e.g., water storage practices) [22,23]. Most literature regarding the simulation of infectious diseases focuses on spatially varying metrics of habitat suitability or on the bidirectional effects of human movement and the area of the epidemic spread, but rarely are both considered in tandem. By considering both spatial and temporal predictors and their interactions, this research provides an unprecedented level of detail in studying and predicting vector-borne disease epidemics.

Here, we focus on Colombia as a focal case study because of its differences in elevation and land cover, micro-variation of climate, demographics, and seasonal patterns in climate and human mobility. The country’s publicly available, fine resolution infection case data made Colombia a particularly worthwhile region of study. Colombia has a population of about 50 million people, and the tourism industry is its second largest export, receiving up to 2.3 million annual visitors in the years leading up to the Zika epidemic [24]. Colombia’s annual calendar can be partitioned into overlapping cycles of wet and dry seasons and peak tourism and off tourism seasons that drive distinct cyclical patterns of mosquito occurrence and human mobility. The Colombia Instituto Nacional de Salud (CINS) began surveillance of Zika virus in August 2015 and first detected 9 cases in October 2015 in the Bolivar region [25]. The nation declared the outbreak over in July 2016, with more than 100,000 cumulative symptomatic cases confirmed [26].

The objective of this research is to analyze the interacting effects of disease vector presence and human movement across metapopulations (a group of spatially separate populations that interact with each other) on disease transmission rates and the probability of super-spreader events, with a focus on tourism-related travel. A super-spreader event is defined as an instance in which a single population node (e.g., a municipality) is responsible for introducing the infection to a large portion of the network [27]. A given node can be quantified by its super-spreader capacity (SSC), defined as the expected number of other nodes to which the node in question is responsible for introducing the infection [28]. Specifically, we test the hypotheses that 1) regions with the highest probability of mosquito presence will have more super-spreader events during dry months, when vector mosquitoes are predicted to be more abundant [22,23,29], 2) regions reliant on travel and tourism industries will have more super-spreader events during wet months despite lower mosquito abundance, because they are more likely to contribute to network-level pathogen spread due to increased travel. First, we use a statistical model to estimate the spatially and temporally varying habitat suitability for *Aedes aegypti*, the primary mosquito vector of Zika virus in Colombia. Next, we combine our vector habitat suitability model with human mobility network models to simulate the course of the Zika virus epidemic if it had begun in each month of 2015. Our prediction is that both the fraction of individuals who become infected with Zika virus, and the spatial distribution and frequency of super-spreader events, are profoundly affected by these seasonal factors, with deep implications for epidemic preparedness and risk management.

## Methods

### Study area

Colombia is a tropical nation with a population of about 50 million people, 80% of which live in urban environments. The nation’s equatorial climate lends itself to a variety of ecosystems, from tropical rainforests in the Amazon basin to the high-altitude Andean mountain ranges. Colombia’s capital and most populous municipality, Bogotá, and is home to about 7 million people, although its high elevation and relatively colder climates make it a poor habitat for mosquitoes. The next four most populated cities, Medellín, Cali, Barranquilla, and Cartagena, are located in warmer climates closer to the western coast. They are all popular tourist locales for international travelers and serve as more suitable mosquito habitats. Although Colombia has undergone rapid modernization in the last decade, about a quarter of the population lives under the poverty line, and political and social unrest are frequent. Rural Colombians outside the five major metropolitan regions have poor access to health care and sanitation, increasing their risk of suffering under an epidemic.

### Mobility network generation

**Fig 1:**
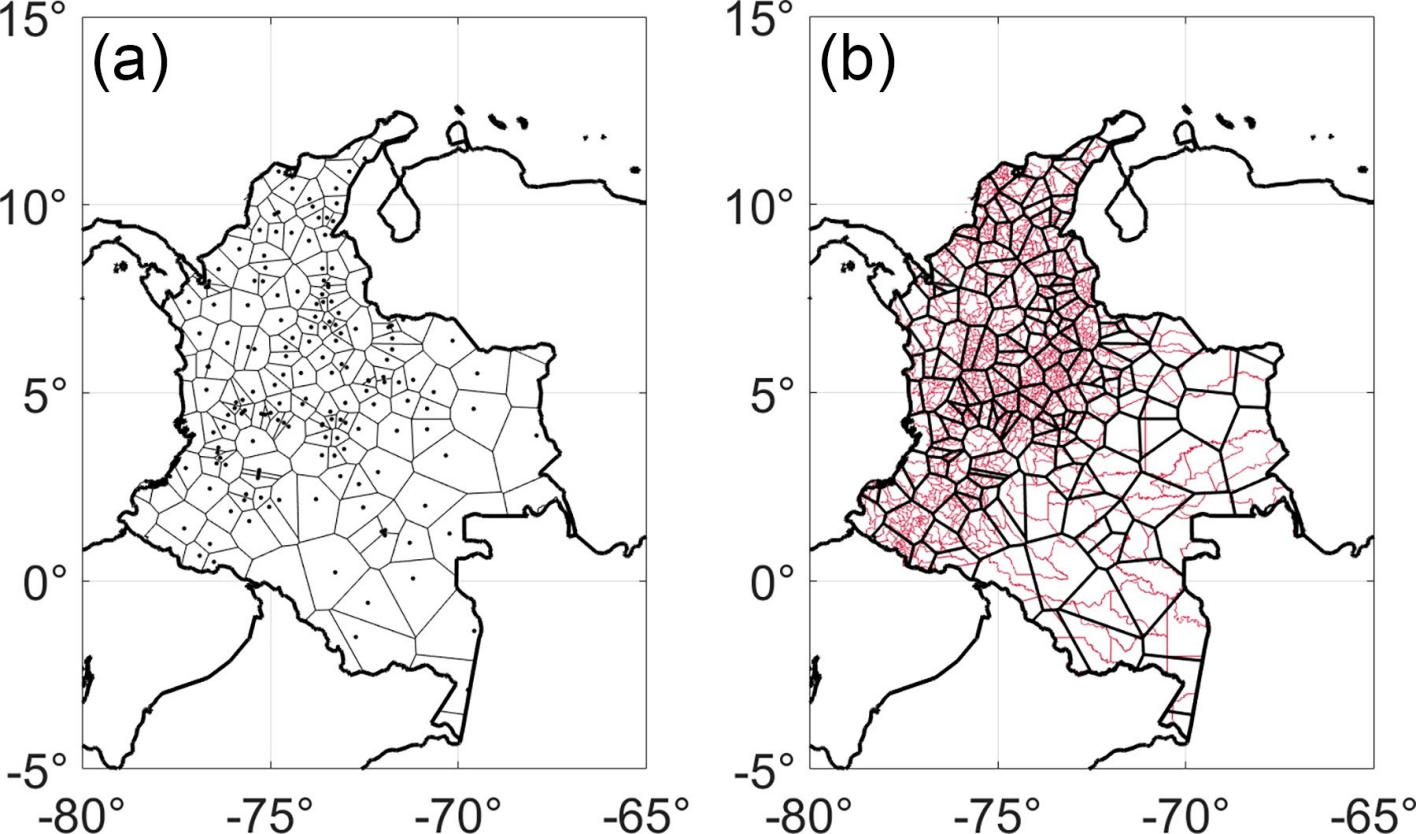
(a) A map of 205 airports in Colombia. The area of study was divided into 205 polygonal regions based on these points using Voronoi tessellation. (b) A map of aggregated regions of human mobility modeled on airport catchments, superimposed over a map of Colombia’s 1123 municipios. Shapefile data was provided by ESRI courtesy of Departamento Administrativo Nacional de Estadística (DANE) and the World Bank [75,76]. Legend: Colombia Municipio Boundaries, https://www.arcgis.com/home/item.html?id=8663c5e0fcfb4556bba049b6c3e5cc60.

To devise a mobility network describing human host movement, 1123 municipalities, or municipios, of Colombia were aggregated into 205 airport catchments based on the locations of all airports and defined using Voronoi tessellation. In a nutshell, Voronoi tessellation converts a series of geographic points into polygonal regions by placing imaginary bubbles at each point, expanding them, and noting where the bubbles intersect [30]. Mobility rates between aggregated regions were computed monthly from 2013 to 2018 from publicly available air travel records provided by the Colombian government [31], which were used to construct the weighted edges of the human mobility network (Fig 1). The raw air travel records provided the total number of passengers traveling between pairs of origin and destination airports, whose values were used to compute the weights for mobility network edges. Airport codes were used to map the boundaries of airport catchments using a multistage process [32]. In the first step, the airport IATA code was translated to geographic coordinates using Google Geocoding API. Voronoi tessellation was calculated from the airport location and used to draw approximate boundaries for each airport catchment. The approximate catchment boundaries were later refined by identifying the municipalities that overlap with each Voronoi airport catchment.

We adopted a population weighted approach to avoid assigning the same municipio to more than a single airport. For example, if the polygon of a municipio overlapped with multiple airport catchments, the overlaps were weighted based on the population count. We estimated the total population count in each area of overlap using the WorldPop unconstrained and UN-adjusted raster of population counts at a spatial resolution of 100 m [33]. Next, the municipio was assigned to the airport with the largest weighted overlap. For cases where multiple airports fell within the boundaries of a single municipio, the travel volumes from each airport were aggregated. Finally, the boundaries of all the municipalities that were assigned to a single airport were dissolved into a single polygon. We defined the airport catchments using municipalities’ boundaries because of the advantages of adding extra socioeconomic attributes that were available at the municipal level.

In total, we collected data from 381,406 domestic flights with approximately 181 million passengers. Our full data set and sources can be found at (https://github.com/a2soliman/Air_Travel_Colombia). Missing data in the mobility network were extrapolated via iterative proportional fitting [34]. This algorithm estimates missing values in sparse matrices based on the marginal distribution of existing data, and for this purpose preserves monthly variation in movement patterns and overall distribution of mobility for each region [35]. Spurious connections with fewer than ten total travelers per month were eliminated from the mobility matrix.

### Environmental-Socioeconomic Maximum Entropy model

We used a Maximum Entropy (MaxEnt) model to estimate habitat suitability for *Ae*. *aegypti*, the primary vector of Zika virus in Colombia. Our model is based on the Boosted Regression Tree model developed by Kraemer (2015) [36], which includes temperature, precipitation, enhanced vegetation index (EVI), and urbanicity as predictor variables. Previous research indicates that *Ae*. *aegypti* thrives based on factors including, in order of importance, long periods of warming between December and January, the minimum monthly temperature, and monthly precipitation [37]. In addition to environmental factors, our model includes several human factors indicating land use and development, socioeconomic status, and aqueduct access (which we assume to be inversely correlated with the prevalence of domestic water storage containers [38,39]). In general, it has been established that Ae. aegypti prefers urban environments, and that poorer communities tend to have higher abundance of disease vectors due to less access to piped water, sanitation and solid waste management services [40]. We curated a set of predictor variables that were each positively correlated with mosquito abundance and were not cross-correlated with each other based on their Pearson correlation coefficient (Table 1). We compiled reported locations of *Ae*. *aegypti* mosquitoes from the Colombia Instituto Nacional de Salud (CINS) and from Kraemer et al. (2015) [36,41], for a total of 886 unique locations where *Ae*. *aegypti* were detected across the country.

**Table 1:** A list of predictor variables used in our MaxEnt model for *Ae*. *aegypti* habitat suitability.

Variable	Description	Resolution	Source
Mean temperature	Mean annual temperature (°C)	15 arcsec	CHELSA [77]
Precipitation	Total annual precipitation (m)	15 arcsec	CHELSA [77]
Elevation	Elevation above sea level (m)	15 arcsec	USGS [78]
Built-up land	Presence of roofed structures (0–1)	15 arcsec	GHS [79]
Land cover	Factorial variable indicating land use (urban, cropland, forest, shrubland, etc.)	15 arcsec	USGS [78]
Enhanced vegetation index (EVI)	Measure of vegetation greenness (0–1)	15 arcsec	USGS [78]
Overcrowding	% of population suffering from overcrowding	Municipio	DANE [80]
Road distance	Distance from nearest highway (m)	15 arcsec	OpenStreetMap [81]
Road density	% of land area occupied by roads	15 arcsec	OpenStreetMap [81]
Poverty	% of population suffering from poverty	Municipio	DANE [80]
Sanitation	% of population with insufficient sanitation	Municipio	DANE [80]
Aqueduct	% of population with access to aqueducts	Municipio	Tufts-Colombia [82]

The MaxEnt model was executed through three generations of hyperparameter optimization, utilizing a genetic algorithm to optimize the Area Under Curve (AUC) value of the Specificity-Sensitivity validation plot [42]. For each generation, 500 MaxEnt models were randomly created and validated, testing every combination of the linear, quadratic, product, and hinge feature classes, and the model with the highest AUC value was used as the basis for the next generation. For our MaxEnt model, we used 60% of the available data for training, 20% for validating each generation, and 20% for testing our final optimized model.

**Fig 2:**
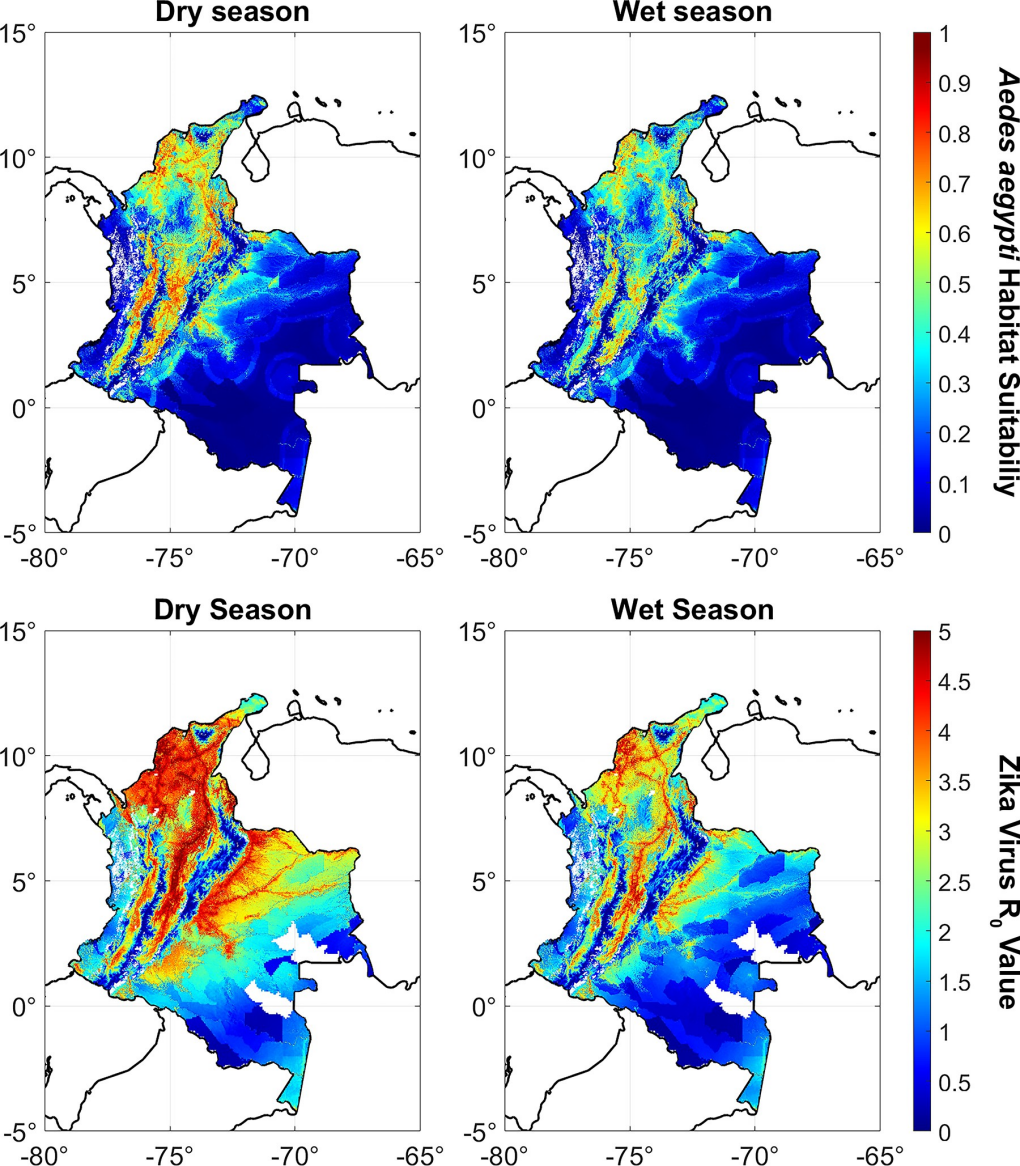
Dry and wet season estimates for (top) habitat suitability for *Aedes aegypti* and (bottom) R_0_ values for Zika virus throughout Colombia. Note that in general we expect much more intense transmission from Zika virus in dry season than wet season, and especially in warm, urban areas. The habitat suitability figures are numbered from 0 to 1, indicating the probability that we would expect to find *Aedes aegypti* at those locations. The Zika virus R_0_ figures are estimates based on habitat suitability, mosquito behavior, and disease transmission and recovery rates using an equation described in the Methods section. An R_0_ value of at least 1 is a necessary condition for an epidemic to occur if the local population is exposed to Zika virus. Legend: base layer, https://datacatalog.worldbank.org/search/dataset/0038272/World-Bank-Official-Boundaries.

**Fig 3:**
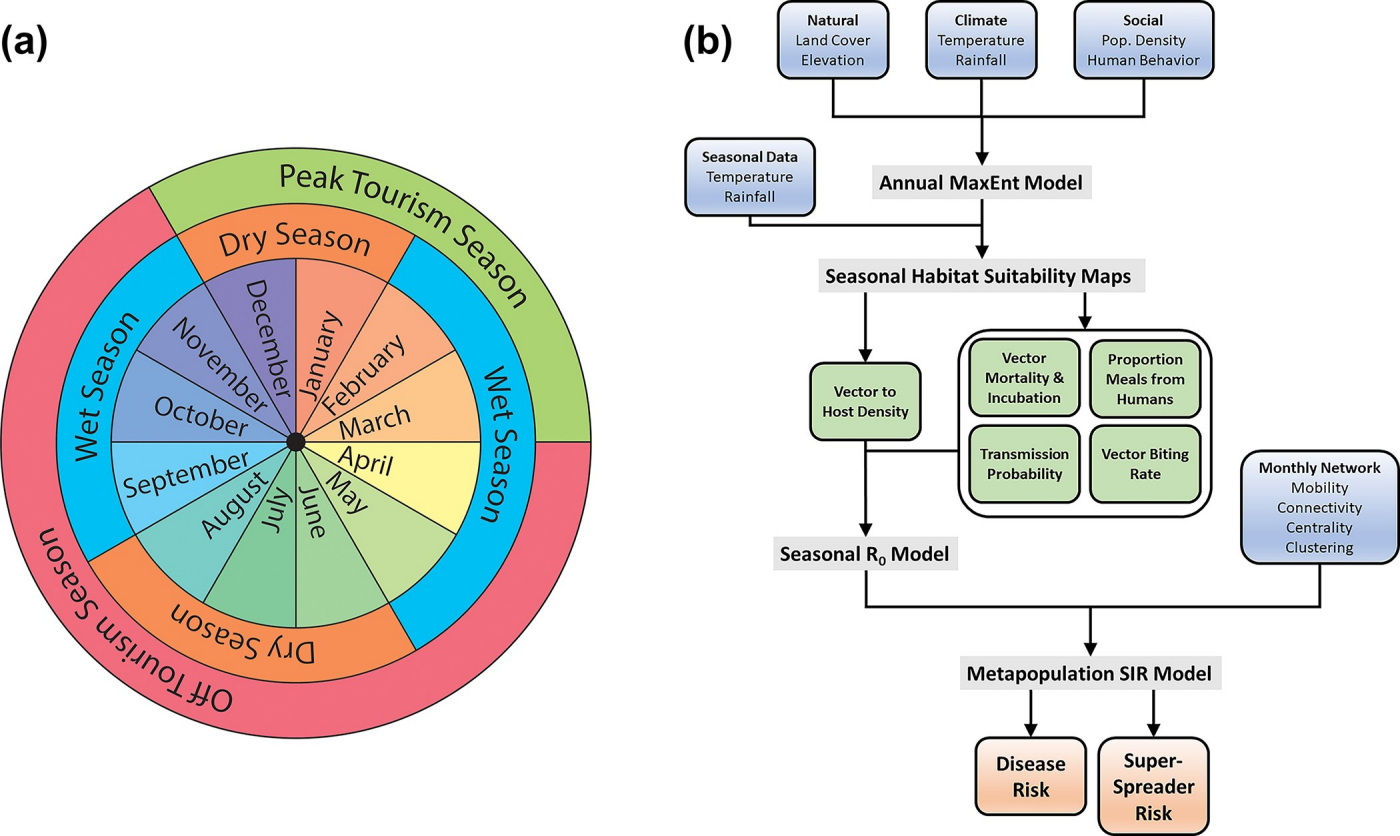
(a) Graphic representation of typical seasonal cycles in Colombia. (b) Flowchart showing the process of data collection, modeling, and simulation used in this study.

After developing a MaxEnt model that computes habitat suitability based on the desired predictor variables, we executed the model again, now using temperature and precipitation rasters with data typical of wet and dry season conditions in Colombia. This produced *Ae*. *aegypti* habitat suitability rasters for the wet and dry seasons (Fig 2). We then used these rasters as predictor variables in the model presented in Caminade et al. (2015) [43], which incorporates weather-dependent mosquito biting rates, vector presences, transmission probability, mortality rates, extrinsic incubation period (EIP), vector to host ratios, and recovery rates to estimate an *R*_0_ value for Zika virus (Fig 3). The formula is adapted as:

$$R_{0}={\left[\left(\frac{b\beta a^{2}}{\mu })(\frac{\nu }{\nu +\mu })(\frac{\phi^{2}m}{r}\right)\right]}^{1/2}$$ (1)

where a is the vector biting rate, b is the vector to host transmission probability, m is vector to host ratio (habitat suitability * 1000), r is Zika virus recovery rate, β is the host to vector transmission probability, μ is mortality rate, ν is Zika virus incubation rate (temperature dependent), and *ϕ* is the human vector preference.

### Metapopulation SIR simulations

The classic Susceptible-Infected-Recovered (SIR) epidemiological model is a mathematical framework to characterize the transmission dynamics of an infectious disease. Individuals from an at-risk population of size *N* are classified among three states (i.e., susceptible; infected; or recovered). Individuals transition from the susceptible to the infected state based upon the transmission rate *β*, and from the infected to the recovered state based upon the recovery rate *μ*. The stochastic SIR model, used in this article, proceeds as follows:

1. The simulation time is incremented by a chosen value *dt*.
2. If at least one individual in the population is infected, a random selection of Susceptible individuals become Infected, as a binomial distribution with probability $1-{\left(1-\frac{\beta *dt}{N}\right)}^{I}$, where *I* is the current number of infected individuals. This reflects the assumption that each individual has an independently distributed probability of becoming infected.
3. Simultaneously, a random selection of Infected individuals become Recovered, as a binomial distribution with probability μ*dt.
4. The simulation repeats, over each time step, until no infected individuals remain.

A metapopulation SIR model is an elaboration of the basic model that considers a network of population centers or nodes, each with its own set of SIR equations, and the rates of population migration between nodes [44]. Individuals migrate from node *i* to node *j* based on a mobility matrix *M_ij_*, which for this study was determined as described above. Daily mobility rates and nodal infection rates were computed from the habitat suitability map and mobility network generated for the month in question. The simulation was set to run for 365 days, with the infection rate map and mobility network being updated each month. The daily infection cases in each region were recorded and used to generate infection curves. We compared our simulation results to empirical case data from Colombia to validate the accuracy of our model by integrating the difference between the simulation and empirical infection curves over time and specifying an ideal relative error of less than 5%. Our empirical dataset of cumulative weekly Zika infections (combined suspected and lab-confirmed cases), reported for each municipio between epi week 41–2015 and epi week 37–2016, was assembled from two sources: the Boletín Epidemiológico and the cdcepi/Zika data repository [25,45].

After validating our algorithm by comparing our generated infection curves to the recorded case data of the 2015 Zika virus outbreak, we executed the simulation 100 times, each with the initial human case introduced in a different month of 2015. In each simulation, we recorded the number of infection cases and the geographic and temporal trajectory of the epidemic, and we statistically compared these metrics to the spatially and temporally varying infection and mobility rates. We focused our analysis on four months in 2015 that are representative of seasonal conditions in Colombia: January, March, June, and September (Fig 3). These months were chosen based on representing seasonal variation in total precipitation (i.e., variation in mosquito habitat suitability) and number of international visitors (i.e., variation in human mobility). Monthly rainfall varies geographically but ranges from 0–5 cm in dry months and 15–20 cm in wet months. Tourism season peaks from December to March in Colombia, when the climate is dry and international visitors arrive in greatest numbers. Tourism season months have upwards of 230,000 international visitors per month, whereas off tourism months usually have about 160,000 visitors [24].

We ran this simulation 100 times, each with a different randomly selected starting location for the epidemic, and statistics such as peak prevalence (maximum fraction of population infected) and super-spreader risk (the rate in nodes per unit time that a given node spreads the epidemic to its neighbors) were averaged over 100 simulations. We then ran a linear regression statistical test comparing the peak prevalence and super-spreader risk of each aggregated region to that region’s nodal centrality, degree of connectivity, mobility rate, *R*_0_ value, and latitude and longitude coordinates. This analysis allowed us to determine which factors were most correlated with epidemic severity and the risk of super-spreader events based on the season.

## Results

### Habitat suitability model results

Our hyperparameter optimized MaxEnt model was able to successfully predict the presence of *Ae*. *aegypti* with an AUC value for data validation of 0.872 (Table 1). In terms of percent contribution to mosquito presence, the most important predictor variables were land cover, road density, elevation, distance to nearest road, and built-up land (Table 2). In general, variables concerning land use and urban development were more highly correlated with habitat suitability than variables concerning climate or infrastructure (e.g., overcrowding, housing availability, and aqueduct access).

**Table 2:** Importance ranking of predictor variables in MaxEnt model for *Ae*. *aegypti* habitat suitability. The AUC value for data validation was 0.872.

Rank	Variable	% Contribution	Permutation Importance
1	Land cover	37.1	24.4
2	Road density	17.3	0.8
3	Elevation	14.2	18.6
4	Road distance	8.8	23.8
5	Built-up land	7.8	17.2
6	Precipitation	6.6	9.8
7	Overcrowding	5.6	2.9
8	Aqueduct	1.0	0.8
9	Poverty	0.7	0.7
10	Sanitation	0.4	0
11	Mean temperature	0.4	0.3
12	EVI	0.2	0.6

To develop wet and dry season habitat suitability models, we used our MaxEnt model to run a prediction algorithm, substituting in precipitation and temperature variables for each month of the year 2015. In general, our models predicted significantly higher *Ae*. *aegypti* abundance in dry than wet season, with an average habitat suitability of 0.31 and 0.17, respectively (Fig 2). The greatest difference in habitat suitability between seasons was along the northern portions of the Andes mountain range in western Colombia. This range includes the areas of the country with the highest elevations, coldest temperatures, lowest precipitation, and highest population density. In the wetter, sparsely population Amazon region in the southeast, there was little detectable difference in habitat suitability between seasons.

These seasonal differences in habitat suitability resulted in relatively similar differences in R_0_ values for Zika virus between seasons (Fig 2). The regions where R_0_ was greater than 1, the threshold for an epidemic outbreak, were largely similar between seasons. However, in the event of an outbreak our models predicted that Zika virus would spread much faster in dry season than wet season, with average R_0_ values of 2.2 and 1.5, respectively. The maximum R_0_ values in both seasons was about 5.3, located in vicinity of Cartagena along the Caribbean Sea.

### Airport catchment results

We identified the geographic coordinates of the airport codes in this dataset using Google Geocoding API. We first matched the airport codes to the IATA records that contain the codes and coordinates of the airports. Our initial search resulted in 30,139 matches out of 32,332 flights, and we ran Google’s Geocoder on the remaining 2,193 trips to identify their coordinates using the trip city names. The 1,123 municipalities of Colombia were aggregated into 205 airport catchments based on our approach. Mobility rates between aggregated regions were computed on a monthly basis from 2013 to 2018 from the air travel data. In total, we collected data from 381,406 domestic flights with ~181 million passengers [46].

### Validation and seasonal comparison

Our simulation of the Zika virus epidemic beginning in October 2015 performed well in comparison to the empirical case data. Our simulation predicted ~109,000 confirmed Zika virus cases throughout the epidemic, as compared to ~103,000 reported cases. Among aggregated regions, the root-mean-square of peak prevalence indicated a 0.34% relative error in estimating total cases. Peak prevalence in simulated versus empirical cases were linearly correlated with *R*^2^ = 0.24. In terms of the trajectory of the epidemic, the simulation accurately predicted that approximately half of the regions would be affected by week 15 of the epidemic and almost all regions would be affected by week 25, also when several of the most populated regions would reach their peak infection rates [26].

**Fig 4:**
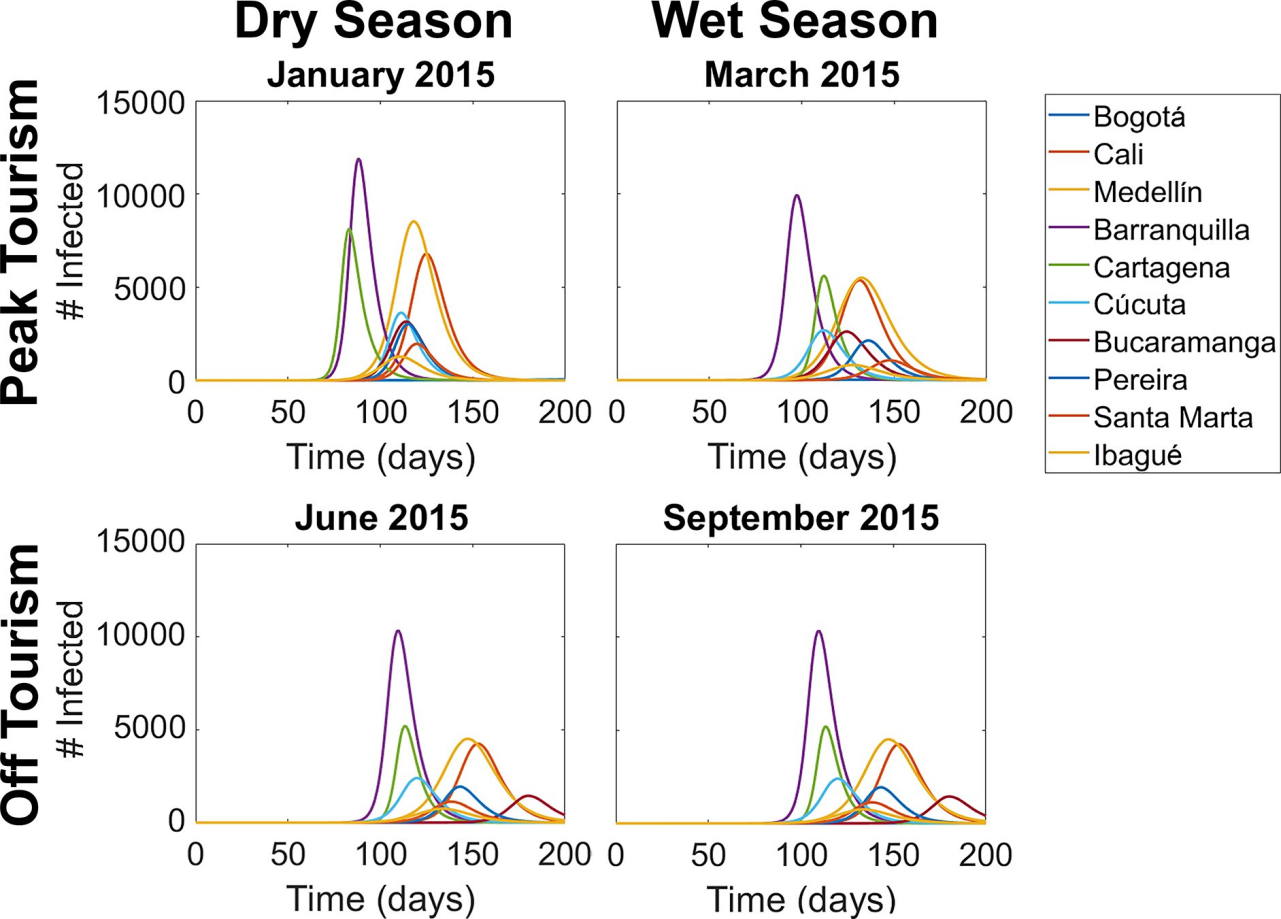
Simulations of Zika virus outbreaks in Colombia if they had started in different months of 2015 corresponding to dry versus wet seasons and peak versus off tourism, with infection curves for the ten most populous cities shown.

We then simulated the conditions that led to the Zika virus epidemic in Colombia, by introducing nine infected humans to the Bolivar region, each in different months of 2015 (Fig 4). These simulations show that if the epidemic had started in January instead of October, the total number of infection cases may have been increased by 60%. In general, epidemics that began in dry months tended to have higher peak prevalence than epidemics that began in wet months. Epidemics that started during peak tourism season tended to spread much more quickly, and the infection curves for major cities tended to have shorter durations. In all scenarios, the city with the most infection cases was Barranquilla, with case rates of 0.75% to 1.1% (9,000 to 13,000 total cases). For other major cities, such as Cartagena, Cali, and Medellín, both the number of infection cases and the timing of the epidemic invasion were changed substantially by the starting month of the epidemic. In peak tourism season, the epidemic spread much faster to regions along the coast with larger travel and tourism industries, whereas in off tourism season the epidemic tended to spread across the country more evenly. Our results indicate that only the dynamics from the first couple months of the epidemic had much effect on its overall trajectory. Once the pathogen was established, the habitat suitability and mobility conditions of future months did not significantly affect the shape of the infection curves.

Our statistical tests indicate that in all seasons, as expected, the most important predictor for peak prevalence is the region’s *R*_0_ value, a function of vector presence (Table 3). Once an infection has been introduced to an area, human mobility in the form of travel and tourism and network structure play a minor role in intrapopulation transmission. However, the factors that predict the risk of super-spreader events vary considerably by season. On average, the month of January had the largest number of super-spreader events per simulation (10.8±1.4), March and June had approximately the same average number of super-spreader events (8.7±1.2) and (8.9±1.2), and September had the fewest (7.4±1.1). This indicates that either a dry season or an increase in travel and tourism-related mobility increases the frequency of super-spreader events. In all seasons, the most important factor for predicting super-spreader risk was network centrality. Human mobility rate in the form of travel and tourism was a statistically strong factor only during the peak tourism season, and the *R*_0_ value was statistically relevant in every month except January.

**Table 3:** F-test results of a statistical test between peak prevalence and super-spreader capacity, on the aggregated municipio level, with nodal centrality, degree of connectivity, outward mobility, R_0_ value, and geographical coordinates as predictor variables. Variables with statistically significant correlations are shown in bold.

Peak Prevalence	January 2015		March 2015		June 2015		September 2015	
Mean	0.16±0.01		0.12±0.008		0.16±0.01		0.11±0.009	
	F value	p value	F value	p value	F value	p value	F value	p value
centrality	1.6	0.211	2.5	0.119	0.1	0.770	0.5	0.494
degree	**12.3**	**0.001**	**6.9**	**0.009**	3.6	0.057	**9.4**	**0.003**
Mobility	5.0	0.027	**7.7**	**0.006**	5.7	0.017	3.5	0.061
R_0_	**97.2**	**<0.001**	**217.6**	**<0.001**	**78.1**	**<0.001**	**102.6**	**<0.001**
Longitude	1.4	0.238	0.0	0.938	4.4	0.037	0.0	0.873
Latitude	1.1	0.306	0.7	0.396	0.3	0.569	0.0	0.959
Super-Spreader Capacity	January 2015		March 2015		June 2015		September 2015	
Mean	13.5±1.6		8.7±1.2		11.2±1.3		7.4±1.1	
	F value	p value	F value	p value	F value	p value	F value	p value
Centrality	**63.2**	**<0.001**	**51.4**	**<0.001**	**59.1**	**<0.001**	**27.0**	**<0.001**
Degree	**7.4**	**0.008**	**10.0**	**0.002**	**5.0**	**0.010**	**9.7**	**0.002**
Mobility	**12.6**	**0.001**	**9.1**	**0.003**	2.1	0.149	0.1	0.794
R_0_	4.9	0.030	**23.9**	**<0.001**	**6.2**	**<0.001**	**24.2**	**<0.001**
longitude	2.3	0.134	1.7	0.191	4.0	0.047	1.4	0.243
latitude	0.3	0.566	3.6	0.060	1.8	0.183	2.0	0.161

## Discussion

This discussion is divided into four parts: habitat suitability of vector species, mobility of human hosts, super-spreader risk, and further discussion of the implications and limitations of this research. We discuss the numerical results of our research as well as its broader implications for vector-borne epidemic forecasting and prevention.

### Implications of habitat suitability

Our research of the well-studied case study of Colombia demonstrates that the severity of a potential Zika virus epidemic varies significantly based on overlapping seasonal cycles of mosquito vector habitat suitability and human mobility patterns in relation to the travel and tourism industry [47–49]. In addition, the risk of super-spreader events varies in magnitude and in spatial distribution depending on these seasons. A Zika epidemic that begins in a dry, peak-tourism month like January may have up to 60% more infection cases than one that begins in a wet, off tourism month like September. In terms of peak prevalence, the total fraction of a subpopulation that becomes infected over the course of the outbreak, the seasonality of mosquito habitat suitability matters much more than human host mobility as related to travel and tourism. Additionally, our simulations indicate that only the first couple of months of the epidemic influence its trajectory, and once the virus is introduced to a subpopulation node the infection curve will be largely determined by the local infection rates. However, our statistical tests show that each municipio’s degree of connectivity within the network is an important predictor for its peak prevalence, even when accounting for the relationship between network connectivity and total population (Table 3). In practice, determining retrospectively when an epidemic begins may be difficult, especially for a typically asymptomatic diseases like Zika virus [50], and the ease of forecasting an epidemic may itself be seasonal [51]. Future research may benefit from the use of neural networks to further elucidate the relationships between network structure and epidemic trajectory [52].

The results of our habitat suitability estimates for *Ae*. *aegypti* indicate that socioeconomic predictor variables, especially those related to land use and road density, should not be neglected in model development [53]. Based on percent contribution to vector abundance, four out of the five more important variables used in our model were related to land use, with elevation being the only statistically significant natural factor [54,55]. On the other hand, the traditional predictor variables of temperature, precipitation, and enhanced vegetation index were relatively unimportant after controlling for other variables. Temperature does tend to be inversely correlated with elevation, but comparing these two variables directly shows that elevation is a much stronger predictor of vector abundance than annual mean temperature. Other factors related to quality of life, such as overcrowding, housing availability, and aqueduct access, were also not statistically significant in this study. These results highlighting the significance of land use are consistent with other habitat suitability studies [56–58], however variables indicating land development may have been confounded by other factors, since in Colombia urban districts are generally wealthier than rural districts. Even though precipitation was not a strong predictor variable, the difference in rainfall between wet and dry seasons is so substantial that we still observed considerable variance in habitat suitability between the seasons.

### Implications of host mobility

Host mobility was determined to be an important factor for the area of the epidemic spread, or the total number of nodes that become exposed to the infection. In addition, the epidemic spread considerably faster during peak tourism season than off tourism season. On average it took about 52 days for the global infection curve to become exponential in peak tourism season, as opposed to 76 days in off tourism season. The infection curves were on average about 20% narrower during peak tourism season than off tourism season, in terms of the time before a subpopulation reached peak prevalence. In other words, during tourism season we expect the epidemic to spread to new locations more rapidly, and once the disease enters an uninfected area it will establish itself in a much shorter time frame [59].

Although incorporating human mobility data related to travel and tourism in studying the spread of diseases is advantageous, the availability of human mobility data at subnational levels in many countries is either lacking at a temporal scale relevant to the study of the spreading of diseases [60] or restricted in availability [61]. Movement of people to and within Colombia has been driven by conflict-induced internal displacement associated with the armed conflict, drug cartel activity, natural disasters, and tourism [62]. Interregionally displaced refugees from the war primarily relocated to cities and border towns. The number of Internally Displaced People peaked in 2022. According to Rengifo-Reina et al. (2023) [63], over 30% of the population in Colombia has experienced forced migration within the country due to the internal conflict, economic reasons or natural disasters. Furthermore, political and economic instability in neighboring Venezuela has created heavy immigration, especially to focal points like Bogota, Medellin and various northern border towns such as Santander [64,65]. It has been estimated that around 1.8 million Venezuelan refugees currently reside in Colombia [66]. In the case of Colombia, Sorichetta et al. [67] used internal migration patterns in malaria-endemic countries, including Colombia, as a proxy for human mobility at the Department levels. While Siraj et al. [68] combined the internal migration data with a geospatial model to down-scale the migration record to a finer geographic scale at the municipal level. Nevertheless, the temporal resolution of migration data remained coarse and represented the slow pace of long-term mobility patterns within the country. More recently Perrotta et al. [61] have used phone data records from a single provider in Colombia to enhance both the spatial and temporal resolution of monitoring human mobility, although the dataset captures a fraction of the population and is available under different restrictions. Integrating these multi-scale dynamics into a metapopulation SIR simulation would be difficult, since such simulations generally function on time frames of at most a few months, however it would be worthwhile to consider the possibilities of migration events as new entryways for infectious diseases [69].

### Seasonal variation and super-spreader risk

**Fig 5:**
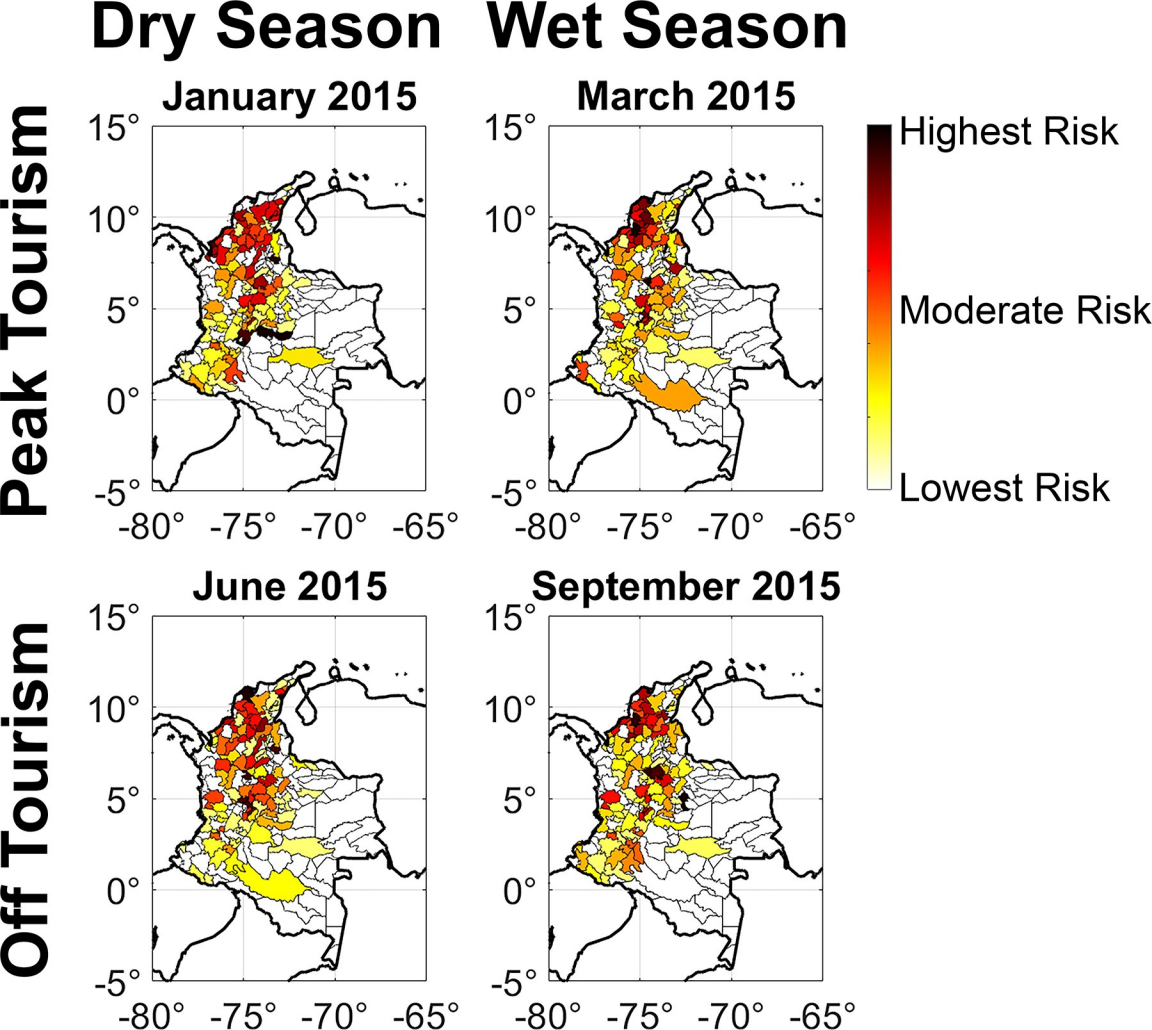
Super-spreader risk map of Colombia for each of four seasonal simulations corresponding to dry versus wet seasons and peak versus off tourism. Risk is quantified by the natural log of the super-spreader capacity as defined in the Methods section. Specifically, on the condition that a given region is exposed to Zika virus, the super-spreader capacity is the expected number of neighboring regions that the epidemic will spread to through host movement. This is measured through a timeline analysis of 100 metapopulation SIR simulations where the initial conditions are slightly varied each time. Each subfigure is calibrated so that the risk level covers the length of the color bar. Legend: base layer, https://datacatalog.worldbank.org/search/dataset/0038272/World-Bank-Official-Boundaries.

The overlapping seasons of vector habitat suitability and human host mobility related to travel and tourism had a significant effect on the frequency and magnitude of super-spreader events (Fig 5) [26,70,71]. In each simulation, there were typically between 25–30 severe super-spreader events in dry months, compared to 15–20 severe super-spreader events in wet months, in which a single municipio spread the Zika virus infection to at least ten neighboring nodes. In peak tourism season, a given municipio’s super-spreader capacity was highly correlated with the total amount of human hosts entering and exiting the node, whereas in off tourism season, the area’s local *R*_0_ value was a most important predictor (Table 3).

It is difficult to retrospectively determine the locations of super-spreader events and the trajectory of the epidemic from empirical case data. However, based on the chronological order in which Zika virus was introduced to municipios, we can infer that popular tourism destinations near the Caribbean Sea with high habitat suitability, such as Cartagena and Medellín, were high risk locations for super-spreader events. The precise details of super-spreader events depended highly on somewhat arbitrary simulation parameters, so it’s best to focus on general regions of the network map rather than specific nodes. That said, in our January and March simulations, the highest magnitude super-spreader events occurred in smaller municipios that lie on the Valle de Magdalena between Cartagena and Bogotá, such as Manaure, Bosconia, and Acacias. In our June and September simulations, the super-spreader capacity was more correlated with regions of high vector habitat suitability, such as the regions near the Caribbean Sea and east of the Cordillera Oriental mountain ranges. These results offer a new dimension to the study of super-spreaders, indicating that their location and severity may vary seasonally, and this line of inquiry merits further research to test its reproducibility to other countries and other infectious diseases.

### Limitations and future research

The epidemiological model used in this research makes several assumptions and simplifications to account for limitations in available data. For example, the wet and dry season habitat suitability maps were trained by the same *Aedes aegypti* site data, as the data did not identify the month of each sighting. The metapopulation SIR model does not distinguish between varying infection and mobility rates among different demographics, such as age or income, and the model does not recognize which individuals are travelling for tourism and who is travelling for work or other reasons. An agent-based model that considers every individual in Colombia as a unit of computation may overcome some of these issues, but the computational expenses would be enormous. This research handled gaps in the available data through interpolation, but future research may be able to take advantage of artificial intelligence (AI) to achieve more thorough results [72–74]. Ideally it will be possible to input a patchwork of airline and road data into an AI-driven code and produce an intricate and detailed mobility model for future simulations. AI could also help streamline the methods used in this manuscript to make our research results easier to reproduce to other nations and diseases.

Because arbovirus transmission is inherently a socio-ecological process, efforts to model the contributions of human mobility and vector habitat suitability to the occurrence of human cases offers considerable potential to improve vector control and disease management. Here, we show that super-spreader events for Zika virus in Colombia were most like to occur in dry months and during periods of peak tourism, due to increased vector habitat suitability and human mobility, respectively. Considering the capacity for *Aedes* spp. mosquitoes to transmit several arboviruses that could be introduced due to global human travel, vector control and public health efforts should focus on these conditions that maximize potential for pathogen establishment and geographic spread. Other mobility patterns related to seasonal migration, drug-trafficking activity, refugee movement, may have also played an important role in epidemic spread and should not be neglected in future studies.

## Conclusion

Our Zika virus epidemic model demonstrates that there are significant benefits to considering both environmental and human factors when estimating habitat suitability for disease vectors and for simulating disease outbreaks, specifically the ability to make fine scale predictions to regions of interest and to predict the effectiveness of mitigation strategies such as environmental controls and movement restrictions. In countries with a high economic reliance on tourism and heavy traffic of international and domestic visitors across regions, especially in the Caribbean and Latin America, an examination of annual variation in climate and human mobility patterns is necessary to accurately estimate the prevalence and spread of outbreaks, as these factors can vary strongly depending on the time of year when the epidemic begins. The risk factors that determine peak prevalence and the risk of super-spreader events vary based on these seasonal patterns. Replicating the methods used in this research, including the predictors for the habitat suitability model and the mobility data used in simulations, may initially be difficult to replicate across other nations, as each country has different policies regarding the dissemination of demographic and infection case data. In the future, machine learning and artificial intelligence may provide a valuable tool to overcome these challenges. Ideally this research can help develop an algorithm that can provide policy makers with a straightforward and precise means of forecasting the dynamics of theoretically epidemics, predicting which cities are at greatest risk of severe infection rates and superspreader events. This process would aid future planning and resource allocation for disease control that can adapt rapidly depending on the location and time of the outbreak.

## Supporting information

S1 Text Data sources. (DOCX)
