# Supplementary material for: The effects of seasonal human mobility and Aedes aegypti habitat suitability on Zika virus epidemic severity in Colombia
Source: PLoS Negl Trop Dis. 2024 Nov 6;18(11):e0012571. doi: 10.1371/journal.pntd.0012571 (PMC11540183; doi:10.1371/journal.pntd.0012571)
Supplement: S1 Text — (DOCX) [file pntd.0012571.s001.docx]

# Supplementary file on data sources used in manuscript

- Colombian municipal border and international border shapefile data was provided by ESRI courtesy of Departamento Administrativo Nacional de Estadística (DANE) and the World Bank.

Bauer M. Colombia Municipio Boundaries. ESRI. https://www.arcgis.com/home/item.html?id=8663c5e0fcfb4556bba049b6c3e5cc60. Published 2024.

World Bank Official Boundaries. World Bank. https://datacatalog.worldbank.org/search/dataset/0038272/World-Bank-Official-Boundaries. Published 2024.

- *Aedes* mosquito siting data was derived from Colombia Instituto Nacional de Salud and *Kraemer, et al.* (2015)

Kraemer MUG, Sinka ME, Duda KA, et al. The global compendium of Aedes aegypti and Ae. albopictus occurrence. Sci Data. 2015;2. doi:10.1038/sdata.2015.35

Dirección Redes en Salud Pública Grupo Entomología. Vectores de Dengue –Chikungunya, Estado Actual.; 2014. https://www.minsalud.gov.co/sites/rid/Lists/BibliotecaDigital/RIDE/IA/INS/Zika-vector-22-mayo-2015-entomologia-vector.pdf.

- Human mobility data within Colombia was provided by the Colombia government.

Especial ACUA. Aerocivil Home Page. <https://www.aerocivil.gov.co/> . Published 2023. Accessed January 8, 2023.

- Zika virus case data was sourced from the Boletín Epidemiológico and the cdcepi/Zika data repository

Salud IN de. Colombia National Institute of Health. Number of confirmed and suspected zika cases by municipality. <https://www.ins.gov.co/buscador-eventos/Paginas/Vista-Boletin-Epidemilogico.aspx>. Published 2020. Accessed January 8, 2023.

Center of Disease Control and Prevention U. Data repository of publicly available Zika data. Github Repository. https://github.com/cdcepi/zika. Published 2016. Accessed January 8, 2023.

- Mean temperature and precipitation data were provided by CHELSA.

CHELSA. CHELSA – Free climate data at high resolution. https://chelsa-climate.org/. Published 2023. Accessed January 8, 2023.

- Elevation, land cover, and enhanced vegetation index data were provided by USGS.

USGS. USGS Science Data Catalog (SDC). US Geological Survey. <https://data.usgs.gov/datacatalog/>. Published 2019. Accessed January 8, 2023.

- Built-up land data were provided by GHS.

European Commission - Joint Research Center. GHSL - Global Human Settlement Layer - Data. Ghsl R2022a. https://ghsl.jrc.ec.europa.eu/download.php. Published 2023. Accessed January 8, 2023.

- Socio-economic data on Colombia were provided by DANE.

Departamento Nacional de Planeación. Colombia, Potencia de la Vida-Fin de la Pobreza. https://ods.dnp.gov.co/es/objetivos/fin-de-la-pobreza. Published 2023. Accessed January 8, 2023.

- Road data were provided by OpenStreetMap.

OpenStreetMap Contributors. OpenStreetMap. http://www.openstreetmap.org. Published 2020.

- Aqueduct data in Colombia was provided by Tufts-Colombia.

Tufts-Colombia. Aqueduct Coverage Per Municipality, Colombia, 2011. https://maps.princeton.edu/catalog/tufts-colombia-aqueductcoveragetotal-11. Published 2011. Accessed January 8, 2023.

Legend: ESRI, Environmental Systems Research Institute; DANE. National Administrative Department of Statistics; ACUA, Colombian Civil Aviation Authority; CHELSA, Climatologies at high resolution for the earth's land surface areas; USGS, United States Geological Survey; GHS, Global Human Settlement
